# Retroviral Infections in Sheep and Goats: Small Ruminant Lentiviruses and Host Interaction

**DOI:** 10.3390/v5082043

**Published:** 2013-08-19

**Authors:** Amaia Larruskain, Begoña M. Jugo

**Affiliations:** Genomics and Health Group, Department of Genetics, Physical Anthropology and Animal Physiology, Faculty of Science and Technology, University of the Basque Country UPV/EHU, Bilbao 48080, Spain; E-Mail: amaia.larruskain@ehu.es

**Keywords:** small ruminants, lentivirus, Visna/Maedi, caprine arthritis encephalitis, host genetics

## Abstract

Small ruminant lentiviruses (SRLV) are members of the Retrovirus family comprising the closely related Visna/Maedi Virus (VMV) and the Caprine Arthritis-Encephalitis Virus (CAEV), which infect sheep and goats. Both infect cells of the monocyte/macrophage lineage and cause lifelong infections. Infection by VMV and CAEV can lead to Visna/Maedi (VM) and Caprine Arthritis-Encephalitis (CAE) respectively, slow progressive inflammatory diseases primarily affecting the lungs, nervous system, joints and mammary glands. VM and CAE are distributed worldwide and develop over a period of months or years, always leading to the death of the host, with the consequent economic and welfare implications. Currently, the control of VM and CAE relies on the control of transmission and culling of infected animals. However, there is evidence that host genetics play an important role in determining Susceptibility/Resistance to SRLV infection and disease progression, but little work has been performed in small ruminants. More research is necessary to understand the host-SRLV interaction.

## 1. Small Ruminant Lentiviruses (SRLVs)

Lentiviruses are a subgroup of the Retrovirus family, which infect a wide range of species including humans and livestock species, causing slow infections that may lead to progressive degenerative diseases. Visna/Maedi Virus (VMV) and Caprine Arthritis-Encephalitis Virus (CAEV) infect sheep and goats. Phylogenetic analyses have established that they are part of a viral continuum and therefore are grouped together as Small Ruminant Lentiviruses (SRLVs) [1,2,3].

### 1.1. SRLV Infection and Disease

Although SRLVs can infect a variety of cells, their target cells are of the monocyte/macrophage lineage where viral expression is closely dependent on the monocyte to macrophage maturation [4,5,6,7].

SRLVs are mainly transmitted horizontally or from dam to offspring via the respiratory and lactogenic routes. Both asymptomatic and diseased animals are a source of viruses for naive animals [8,9,10,11,12,13,14,15,16].

**Figure 1:**
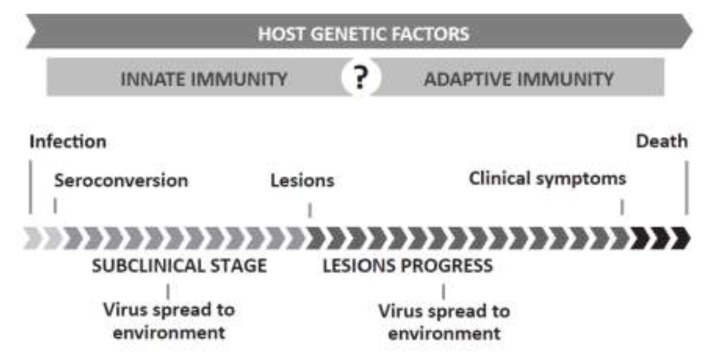
Small Ruminant Lentivirus (SRLV) timeline. SRLVs are transmitted via the respiratory or lactogenic route. Proviral DNA transcription and gene expression is suppressed until infected monocytes mature into macrophages. Once in the target organs, infected macrophages initiate viral replication, which induces an inflammatory cascade. Despite a vigorous immune response, the virus is not cleared and the animal will remain a carrier for life. Most animals remain asymptomatic, but a certain percentage of the animals develop the clinical disease in one or several target organs. Subclinical infection and disease progression may range from months to years. Both Visna/Maedi (VM) and Caprine Arthritis-Encephalitis (CAE) are ultimately fatal. Host genetic factors, infecting viral strain and management influence the occurrence, length and spectrum of affected target organs.

Lesions induced by SRLVs are characterized by mononuclear infiltration in target organs and persistent inflammation [17,18]. SRLVs cause Visna/Maedi (VM) in sheep and Caprine Arthritis-Encephalitis (CAE) in goats, multisystemic inflammatory diseases affecting lungs, the central nervous system (CNS), joints and mammary glands. In both cases, symptoms take several months to years to develop (see Figure 1 for disease timeline). The syndromes may be present to varying degrees in the same animal or occur independently [6,10,19,20,21,22].

### 1.2. Relevance and Control of SRLVs

SRLV infection and associated diseases are present in most sheep-rearing countries worldwide, negatively affecting production and animal welfare [1,17]. The economic impact depends on the breed, management system and type of production [6,10,11,23].

SRLV infections are difficult to control due to the ability of the virus to integrate into the host’s genome, induce persistent infections and elude neutralization. Vaccination attempts have occasionally caused increased viremia and more severe disease [1,6,18,24,25,26,27,28,29]. Currently, the control of VM and CAE relies on the control of transmission and test and cull schemes of infected animals and their progeny [30,31,32].

The development of disease-resistant breeding stock has been undertaken in other diseases due to lack of effectiveness of vaccines and development of resistance of pathogens to drugs and chemicals, which are becoming increasingly common [33]. However, the detection of SRLV-resistant animals remains problematic since DNA markers have to be identified and the interaction of SRLVs with the host and the pathogenesis of VM and CAE are not fully understood. It is therefore necessary to dissect the antigen and immune response if appropriate control methods are to be developed [34].

## 2. Host Genetic Resources

Since the domestication of sheep and goats human-mediated breeding has generated specialized animals suitable for a wide range of purposes, and hundreds of breeds exist worldwide which often have their own genetic signature [35,36].

There has been a huge increase in the research and application of disease genetics in small ruminants due to the growing knowledge of the influence of host genetics on disease control and the increase in available genetic and genomic tools. Apart from the scientific interest, there is a growing pressure on breeders to select animals that are healthier and more resistant both to infectious and metabolic diseases, which could be feasible with the available biotechnology tools and quickly augmenting knowledge on lentivirus molecular biology/pathogenesis [32,33].

However, in general the development of small ruminant genetic and genomic resources lags behind that of other domestic species such as cattle [37,38,39].

Different markers and strategies can be used to discover disease-associated loci, including microsatellites, single nucleotide polymorphisms (SNP), copy number polymorphisms or variants, *etc.*, which in addition can be complemented with expression assays [38,40]. Current high-throughput techniques allow the rapid genotyping of markers such as SNPs. (see Table 1 for limitations of association studies).

## 3. Host-Viral Pathogen Interaction

The interaction between retroviruses (RV) and their hosts is complex. RVs are obligate pathogens that require the host’s cellular machinery to complete their cycle. In response, the hosts have developed sophisticated mechanisms to fight viral infections, including innate and adaptive immune systems [50,51]. Observation of retroviral diseases in various species has shown that even in a population infected by a particular virus strain there are usually differences in the outcome of infection and disease of individual animals [52], and studies on host immune response, gene variability and expression could reveal why.

In humans, host genetic factors are important determinants that influence susceptibility to HIV infection and subsequent progression to AIDS, which has been thoroughly studied. Inter-individual variability in susceptibility to HIV-1 infection, transmission, disease progression, and response to antiviral therapy have been attributed to host determinants and variability in multiple genes [53,54].

**Table 1:** Limitations of genetic association studies in complex multifactorial diseases such as SRLV-induced pathologies. Factors leading to confounding that may be encountered as well as their consequences are shown (information compiled from [41,42,43,44,45,46,47,48,49].

HOST			GENOTYPING		
**Population stratification**	Often present in livestock due to breeding practices		**Bias/Partiality**	Case samples are treated with preference	
**Sample size**	Affects power to detect association		**Marker density**	Often few markers are analyzed per gene	
**Phenotype**	Description must be accurate		**Marker frequency**	Frequency of marker variants affect odds of detecting association	
**Age**	Older animals have been exposed for a longer time		**STATISTICAL ANALYSIS**		
**Gene effect**	Genes involved have small/moderate effects		**Power**		Depends on sample size, marker frequency and gene effect
**Presence of other diseases**	May facilitate SRLV pathogenesis		**Gene interaction**		Often unaccounted for
**SRLVs**			**Confounding factors**		Failure to account for them may lead to erroneous interpretations
**Different virus strains**		Different virulence and host/organ spectrum complicates research;	**Multiple corrections**		Necessary yet may lead to reject real associations
		Strain variability may affect detection of infected individuals	**Consistency/replicability**		Results must be replicated in different populations/
**ENVIRONMENTAL FACTORS**					Can results be replicated in a different population?
**Husbandry**		Prolonged and crowded housing enhances infection			

SRLVs share many characteristics with HIV and indeed, in small ruminants, differences in susceptibility have been observed as well. However, a characteristic that differentiates SRLVs from other lentiviruses is that they do not cause immunodeficiency. In fact, one of the unresolved issues in SRLV infections is the fact that the virus is not cleared despite the host mounting an immune response. It is believed that SRLV proviral DNA escapes detection by the immune system by persisting in monocytes, thus allowing viral dissemination to target tissues. Furthermore, SRLVs may interfere with the function of effector cells implicated in the defense against the virus, such as macrophages and dendritic cells, altering the type of immune responses that are induced [5,11,17,55,56].

Other lentivirus species, although not as extensively studied as HIV, have added information on host genetic factors that may influence lentiviral pathogenesis including Equine Infectious Anemia Virus (EIAV), bovine immunodeficiency virus (BIV), Jembrana disease virus (JDV) and feline immunodeficiency virus (FIV). Among these species, SRLVs, EIAV and BIV/JDB have a tropism for cells of the monocyte/macrophage lineage, while the others also infect lymphocytes. In these non-primate lentivirus species differential susceptibility and associated genes may be found as well [1,7,57].

## 4. Evidence Pointing to the Existence of Host Genetics Controlling SRLV Pathogenesis

SRLV-induced pathogenesis is a complex process determined by multiple factors (See Table 1 for more details) such as host genetics, virus strain, management practices and presence of other diseases [10,17,32,58,59,60,61,62,63].

The above factors must be accounted for, but do not rule out genetic susceptibility. There is evidence that host genetics play an important role in determining susceptibility/resistance to SRLV infection and pathogenesis, but little work has been performed in small ruminants [64].

Research indicates that a breed (or genetic) predisposition may exist regarding infection susceptibility, provirus levels and lesion occurrence and progression, and thus the influence of the host’s genetics can be studied at different levels. The VMV-infected asymptomatic Karakul sheep imported to Iceland in the 1930s never developed signs of the disease but their importation resulted in a widespread dissemination of VM to the extremely susceptible native Icelandic sheep [34]. The research carried out by Icelandic scientists showed that some flocks/family lines had a slower disease progression. In addition, progression of Maedi was delayed in Icelandic x Border Leicester cross individuals [17,32,65]. Research carried out in other countries/breeds has provided further evidence of differential individual and breed susceptibility as well [55,66,67,68,69].

### 4.1. Infection

Resistance to SRLV infection despite repeated exposure could be interpreted as an innate resistance to infection [64,70]. Significant breed effects on seroprevalence have been observed in comingled flocks, with some sheep and goat breeds being more likely to seroconvert, indicating a possible host genetic restriction. For instance, a higher seroprevalence has been demonstrated in Texel, Border Leicester and Finnish sheep breeds as opposed to Ile de France, Rambouillet and Suffolk [10,23,32,68,71,72]. Saanen and Toggenburg goats are more likely to seroconvert whereas Bedouin Black could be resistant to infection under natural conditions [73,74].

In Latxa dairy-sheep, seroconversion was strongly associated with lifetime maternal VMV-serological status. Offspring of consistently seronegative ewes (>4 years old) were less likely to become infected, suggesting the existence of an inheritable factor of VMV-infection resistance [31].

Herrmann-Hoesing *et al.* [64] detected breed differences in seroprevalence and provirus levels (Rambouillet had lower values in both characters in comparison with Columbia and Polipay), which could imply mechanisms preventing infection and/or aiding in virus control after infection occurs. Interestingly, a correlation has been observed between virus load and the severity of lesions in both sheep and goats [17,21,26,34,75,76] suggesting that breeds/individuals able to control virus levels may be less likely to develop VM and CAE. After infection, there is an acute viremia followed by an immune response that restricts virus replication to low levels; although the infection is not cleared, virus replication is kept at a low level [77]. Some hosts may be better at regulating the extent of viral gene expression in macrophages and perhaps disease progression [1,64]. Latency appears to be controlled by an interaction of cellular and viral transcription factors that regulate viral RNA expression [32,78,79].

### 4.2. Clinical Disease

Concerning disease progression, it does not progress uniformly in all individuals and rapid progressors have been observed [19,56]. Furthermore, the extent of SRLV-induced lesions and the spectrum of affected organs may depend on the host’s genetics as well as the infecting virus strain [10,80]. Certain breeds seem to be more likely to develop the clinical signs, while others remain in a subclinical infection stage. Coarse wool type sheep may be more susceptible than fine wool type sheep [66]. Border Leicester sheep could be more likely than Columbia to develop SRLV-specific lesions [66,67]. Chios and Awassi sheep seem to be highly susceptible to infection but resistant to the development of the clinical disease [32,74,81]. Interestingly, Assaf sheep, a stabilized Awassi x East Friesian cross, show high SRLV seroprevalence and VM clinical disease occurrence including the neurological manifestation [82].

Further evidence is provided by experiments carried out with isogenic twin lambs, which showed that the host’s genetics influence the extent and severity of SRLV-induced pulmonary lesions [70].

## 5. Host Genetic Factors Involved in SRLV-Induced Pathogenesis

SRLV pathogenesis is difficult to analyze due to the strains, different host species and breeds, differential disease progression and affected organ spectrums. It seems that the relationship between small ruminants and SRLVs is complex and that the pathogenesis is likely induced by a number of genes with small or moderate effects [7,56].

The cellular receptor(s) for SRLVs has not been conclusively identified and therefore that information cannot be exploited yet. Most likely it is a common cell membrane molecule as SRLVs can enter other cells apart from target cells, and thus the receptor does not dictate cell tropism [17,34,83,84]. However, classical VMV and CAEV strains appear to use different receptors [7,85,86]. The mannose receptor (MR) is a putative receptor for SRLVs. Crespo *et al.* [87] characterized the ovine MR nucleotide and protein sequence, and its role in VMV infection is currently being studied.

Both innate and adaptive (humoral and cellular) immune responses are induced by SRLVs and various works have identified immune response loci that could influence resistance/susceptibility to SRLV infection and disease, providing evidence that genetic factors might modulate the outcome. Below are some host genetic factors that have been involved in SRLV infection and disease.

### 5.1. Major Histocompatibility Complex (MHC)

The major histocompatibility complex (MHC) region, a polymorphic multi-gene complex located on chromosome 20 in sheep and chromosome 23 in goats [88,89], has been implicated in SRLV infection and SRLV-induced disease. The MHC Class I and II genes encode receptor glycoproteins that bind and present antigenic peptides to T cells initiating the immune response. The MHC is one of the few polymorphic systems for which it has been possible to establish a functional significance for the observed genetic variation [90]. Its polymorphism and association with infectious disease has been intensely studied in a myriad of diseases with viral etiology, although such studies are scarce in small ruminants.

In the ovine species, VMV infection increases the expression level of the MHC Class II in the lung, the CNS and the synovium [76,91,92,93,94]. In addition, Class I and II polymorphic gene variants have been linked to SRLV provirus levels and disease progression (See Table 2 for more details; [64,95,96]).

**Table 2:** Major histocompatability complex (MHC) genes/variants associated with VM or CAE pathogenesis.

Region	Variant	Typing method/marker	Species/Breed(s) studied	Reference
**Class I**	allele CLA Be7	Alloantisera	Goat/Saanen	[97]
	Allele OMHC1* 205	Microsatellite	Sheep/Latxa	[96]
**Class II**	Alleles DRB1*0403- and DRB1*07012 and various amino acid positions	Cloning and direct sequencing	Sheep/Rambouillet, Polipay & Columbia	[64]
	DRB1*0325	PCR-Sequence-Based Typing	Sheep/Latxa	[95]
	Allele DRB2* 275	Microsatellite	Sheep/Latxa	[96]

The highly polymorphic nature of many loci in the MHC, among which the DR beta 1 (DRB1) gene is a clear example (over 100 alleles identified to date in sheep [64]), makes it difficult to investigate its involvement in SRLV pathogenesis [90]. Genotyping methods with enough resolution are necessary to type MHC loci, and sample sizes are often inadequate to test rare variants. The effect of different MHC alleles remains to be elucidated but it is possible that polymorphisms, depending on the location, may alter the ability of MHC molecules to bind and present antigens, affect the interaction with T cells or elicit differential immune responses [98,99].

### 5.2. Antibody and T Cell Response

SRLV infection induces antibody response, which has been well studied due to its use in SRLV serologic diagnostics [100]. However, neutralizing antibodies are slow to be induced or have low affinity and relatively low titers. A small amount of free viruses combined with the ability to spread from cell to cell means that propagation and spread cannot be fully stopped by antibodies [7,101].

Nevertheless, some antibody responses may be better at controlling SRLV. Data indicates that the antibody response is skewed in SRLV-infected sheep and goats. Only immunoglobulin G1 (IgG1) subtype responses are detected in sheep whilst in goats IgG1 dominates the humoral response [19,102,103,104,105].

Concerning T cells, both T cell proliferative and cytotoxic responses are induced after SRLV infection [7]. However, the CD4^+^/CD8^+^ cell ratio is decreased in the bronchoalveolar fluid (BALF), synovial fluid and cerebrospinal fluid of diseased animals because of higher levels of CD8^+^ cells, which could be correlated with the severity of lung lesions [93,94,106,107,108]. Cytotoxic T lymphocytes (CTLs) appear to be important effector cells, for they may inhibit viral replication but also contribute to lesions through cytokine production or cytotoxicity [34,76,109,110].

In ruminants, IgG1 induces a type-2 T helper cell (Th2) response, while production of IgG2 is indicative of a Th1 response [111]. Interestingly, goats with a Th2-biased CD4^+^ T cell response are known to develop arthritis, but those with a mixed IgG1 and IgG2 response remain without clinical signs, suggesting that anti-SRLV IgG2 antibodies protect against disease induction [7,19,104,112]. In addition, CD80 levels, which favor Th1 cell differentiation, were low in clinically affected sheep compared with asymptomatic and seronegative controls [80].

### 5.3. Cytokines and Receptors

Cytokines are important signaling proteins that act as important mediators of the immune system, which initiate and modulate many processes. Studies in diverse species have revealed that cytokine and cytokine receptor expression levels, polymorphisms and profiles in the host can affect the pathogenesis of virus-induced diseases.

Cytokines seem to have an important role in SRLV induced pathogenesis, including the modulation of the ongoing immune activation in target organ lesions with some cytokines favoring the appearance of lesions. Cytokines induce the recruitment and differentiation of monocytes to macrophages as well, favoring additional infection by attracting target cells and thus creating a vicious circle. Indeed, several cytokines have been found up or down regulated (See Table 3) in VM and CAE, although more data on cytokines are necessary [7,10,19,20,112,113,114,115,116,117,118,119,120].

Although most research has focused on expression, a few works have explored the influence of polymorphisms. Cytokine polymorphisms are known to affect cytokine gene transcription [121,122], or they may affect cytokine function thus influencing the outcome of infection and disease. Chemokine receptor 5 (CCR5) is an example of how polymorphisms could affect SRLV pathogenesis. CCR5 participates in leukocyte recruitment against different pathogens, including ovine lentiviruses. A deletion in this locus causes an almost four-fold reduction in transcription in animals carrying the deletion allele, as well as showing association with a decreased provirus-burden. Considering that monocytes/macrophages are the main target cells of VMV, it has been suggested that a reduced chemotactic ability of CCR5 could result in reduced influx of such cells to the site of infection, which could also slow the rate of cellular infection [120,121,123].

Alteration of cytokine profile has been suggested in both sheep, and goats and it is suspected that SRLVs induce a switch to a Type 2 (Th2 type) cytokine response. Evidence backing this is the up regulation of Type 2 cytokines such as anti-inflammatory cytokines IL4 and IL10 [20,80,102,105].

### 5.4. Innate Immunity and Restriction Factors

The role of innate immunity is gaining interest in the field of lentiviral infections [7]. Genes of the innate immune response are being characterized in small ruminants and their association with SRLV pathogenesis is being tested.

Since the innate immunity senses highly conserved pathogen-associated molecular patterns (PAMPs) via a number of receptors and signaling molecules, polymorphisms in innate immune genes are very likely to affect the host susceptibility to infections. Indeed, such an effect has been observed in a number of species and diseases, and thus genes such as Toll-like receptors (TLRs) and their variation have been suggested to be good candidate prospects in various diseases affecting domestic livestock [124,125].

TLRs are a family of transmembrane signaling molecules that trigger both the innate and the adaptive immune response mechanisms in response to PAMPs, including viral components [50,126].

**Table 3:** Cytokine and cytokine receptor genes implicated in SRLV pathogenesis in sheep and goats.

Gene symbol	Gene	Species	Methods	Analyzed material	Parameter analysed	References
**IL1β**	*Interleukin-1beta*	Sheep	Semiquantitative RT-PCR ^1^	Lung	Clinical disease	[114]
**IL2/IL2R**	*Interleukin-2/Interleukin-2 receptor*	Sheep	Semiquantitative RT-PCR, qPCR ^2^	Lung, PBMCs ^3^, lymph node leukocytes	Infection, Clinical disease	[114,118,120]
		Goat	*In situ* hybridization	Joints	Infection, Clinical disease	[113]
**IL4**	*Interleukin-4*	Sheep	Semiquantitative RT-PCR	Lung	Clinical disease	[114]
		Goat	Semiquantitative RT-PCR	PBMCs	Clinical disease	[20]
**IL6**	*Interleukin-6*	Sheep	RT-PCR	Alveolar macrophages	Infection, Clinical disease	[116]
**IL8**	*Interleukin-8*	Sheep	RT-qPCR, *in situ* hybridization, Northern blot, Semiquantitative RT-PCR	BALF, alveolar macrophages,	Infection, Clinical disease	[115,117,119]
		Goat	*In situ* hybridization	Macrophages	Infection	[20]
**IL10**	*Interleukin-10*	Sheep	Semiquantitative RT-PCR	Lung, alveolar macrophages	Infection, Clinical disease	[114,116]
**IFNy**	*Interferon-gamma*	Sheep	Semiquantitative RT-PCR	Lung	Clinical disease	[114]
		Goat	Semiquantitative RT-PCR, *in situ* hybridization	PBMCs, joints	Clinical disease	[19,113]
**TNFα**	*Tumor Necrosis factor-alpha*	Sheep	qPCR	Lung, udder	Clinical disease	[120]
**TGF-β1**	*Tumor growth factor beta-1*	Sheep	Semiquantitative RT-PCR	Alveolar macrophages	Infection, Clinical disease	[116]
		Goat	*In situ* hybridization	Macrophages	Infection	[20]
**MCP-1**	*Monocyte chemoattractant protein 1*	Goat	*In situ* hybridization	Macrophages, joints	Infection ,Clinical disease	[20,113]
**GM-CSF**	*Granulocyte macrophage stimulating factor*	Sheep	Semiquantitative RT-PCR	Lung, alveolar macrophages	Infection, Clinical disease	[114,116]
		Goat	*In situ* hybridization	Macrophages	Infection	[20]
**CCR5**	*Chemokine (C-C motif) Receptor 5*	Sheep	Cloning & sequencing, qPCR	PBMCs, lung	Infection, Clinical disease	[120,121]

^1^ RT-PCR: Reverse Transcriptase PCR; ^2^ qPCR: Quantitative PCR; ^3^ PBMCs: Peripheral Blood Mononuclear Cells.

Mikula *et al*. [127] suggested that individual susceptibility existed in the Tsigai breed that may be defined by the presence of SNPs in TLR7 and TLR8, which sense double stranded viral RNA. Mutations at the TLR7 locus may affect PAMP binding onto the TLR molecule [127,128]. In addition, these two genes have recently been found to be up regulated in diseased sheep [120]. Furthermore, different Toll-like receptors may activate different signaling pathways, with different biological consequences, and thus merit additional research [50].

In response to retroviruses and other transposable elements, which invade the host’s genome, hosts have developed intracellular defenses known as restriction factors. These proteins block the viral cycle using different strategies, such as directly attacking viral structures or editing viral genetic material during reverse transcription [129,130]. In recent years there has been a notable increase in this research area.

Ovine TRIM5α seems to be able to restrict VMV DNA synthesis [131]. This intracellular factor restricts retroviral replication by interacting with the capsid and preventing uncoating. A recent study has suggested an association between a tripartite motif protein 5 alpha (TRIM5α) polymorphism that inactivates antiviral activity and accelerates AIDS disease progression [56,132]. Whether such an effect is also present in sheep and goats will still have to be investigated.

Research is needed concerning additional innate immunity genes. Apolipoprotein B mRNA-editing enzyme (APOBEC3) proteins, which act against a wide variety of retroviruses including HIV, have been studied in sheep and they could be good candidates for SRLVs [7,130,133,134,135]. A human APOBEC3G variant has been associated with rapid HIV-1 disease progression [50].

Recently, allele and haplotype variants of the transmembrane protein gene 154 (TMEM154) locus have been associated with SRLV infection, which has been proposed as a locus that could be used for genetic marker-based selection [23]. Sheep with the ancestral variant haplotypes of this gene had an increased risk of becoming infected compared with individuals with mutant forms. However, the function of TMEM14 has not been established yet.

## 6. Conclusions

So far, a number of genes implicated in SRLV infection and disease have been identified, but more research is necessary to understand the host-SRLV interaction. A better understanding of loci involved in SRLV infection and pathology and their polymorphism could lead to the identification and selective breeding of naturally resistant animals, thus avoiding culling and reducing viral load and disease development [23,131]. There are encouraging examples where breeding programs have successfully selected animals with enhanced resistance, for example nematodes, mastitis, foot rot and scrapie in sheep; nowadays genetic tools are being employed to aid this selection, as seen in scrapie. However, a critical question will be if there is also a correlation between disease resistance and production traits. Breeding for resistance as a sole objective may not be desirable due to the potential negative impact it may have on other desirable production traits [33,90]. In addition, the molecular characterization of host genetic factors involved in SRLV infection and disease could detect new targets, which could uncover new host pathways leading to opportunities to develop antiviral therapies.
